# Switching between Magnetotactic and Aerotactic Displacement Controls to Enhance the Efficacy of MC-1 Magneto-Aerotactic Bacteria as Cancer-Fighting Nanorobots

**DOI:** 10.3390/mi7060097

**Published:** 2016-05-25

**Authors:** Sylvain Martel, Mahmood Mohammadi

**Affiliations:** NanoRobotics Laboratory, Department of Computer and Software Engineering, Institute of Biomedical Engineering, Polytechnique Montréal, Montréal, QC H3T 1J4, Canada; mahmood.mohammadi@polymtl.ca

**Keywords:** nanorobots, cancer, hypoxia, magnetic field, magnetotactic bacteria

## Abstract

The delivery of drug molecules to tumor hypoxic areas could yield optimal therapeutic outcomes. This suggests that effective cancer-fighting micro- or nanorobots would require more integrated functionalities than just the development of directional propelling constructs which have so far been the main general emphasis in medical micro- and nanorobotic research. Development of artificial agents that would be most effective in targeting hypoxic regions may prove to be a very challenging task considering present technological constraints. Self-propelled, sensory-based and directionally-controlled agents in the form of Magnetotactic Bacteria (MTB) of the MC-1 strain have been investigated as effective therapeutic nanorobots in cancer therapy. Following computer-based magnetotactic guidance to reach the tumor area, the microaerophilic response of drug-loaded MC-1 cells could be exploited in the tumoral interstitial fluid microenvironments. Accordingly, their swimming paths would be guided by a decreasing oxygen concentration towards the hypoxic regions. However, the implementation of such a targeting strategy calls for a method to switch from a computer-assisted magnetotactic displacement control to an autonomous aerotactic displacement control. In this way, the MC-1 cells will navigate to tumoral regions and, once there, target hypoxic areas through their microaerophilic behavior. Here we show not only how the magnitude of the magnetic field can be used for this purpose but how the findings could help determine the specifications of a future compatible interventional platform within known technological and medical constraints.

## 1. Introduction

Hypoxic environments with <0.7% O_2_ (less than 5 mmHg partial pressure of oxygen (pO_2_)) in solid tumors are not only highly heterogeneous but also very difficult to treat [1]. Such hypoxic environments are the result of the rapid proliferation of tumor cells that consume oxygen. This lower oxygen level leads to less efficient radiotherapeutic treatments [2,3] while chemotherapeutic agents cannot diffuse effectively towards the hypoxic cells that are relatively distant from blood vessels [4,5]. As a result, modern cancer therapies are still relatively inefficient at targeting tumoral hypoxic regions.

Recent methods of tumor targeting emphasize the use of nanoparticles (NPs) as carriers using passive or active strategies [6]. Both strategies aim at enhancing tumor selectivity over healthy tissues, which is known to be a major limitation of modern cancer therapies. Passive targeting achieves selective drug targeting to solid tumors by exploiting abnormalities of tumor vasculature. Indeed, passive targeting relies on the extravasation of the NPs at the tumor, taking advantage of the tumor microvascular network. This fact, combined with tumor-limited lymphatic drainage, helps to achieve a selective accumulation of NPs in tumor tissues. The latter is known as the Enhanced Permeation and Retention (EPR) effect [7]. Active targeting, on the other hand, relies on specific ligands attached to the surface of the therapeutic carriers to recognize and bind pathological cells.

Currently, both strategies yield relatively low targeting efficacy due in great part to systemic circulation of the pharmaceutical agents when injected outside the tumor. Indeed, systemic circulation results in a relatively large ratio of toxic agents affecting healthy tissues. The lack of diffusion deeper in the tumoral tissues contributes to reducing the therapeutic effects further. This is attributed to a lack of sufficient flow caused by the Tumor Interstitial Fluid Pressure (TIFP). Because of such TIFP, intra-tumoral injection is typically not an option. Although the dominant transport mechanism for small molecules and oxygen is convection driven by concentration gradients, larger macromolecules, such as proteins and genes from engineered therapies, rely on diffusion. Even agents relying on affinity for binding could benefit from an onboard propulsion system with a sensory-based intelligence capable of influencing the displacement of these agents towards the hypoxic regions in order to maximize the therapeutic efficacy.

In this respect, medical nanorobotics could play a major role in achieving enhanced targeting to specific regions by providing some level of intelligence and navigation assistance from medical imaging modalities. To directly target solid tumors, the addition of propelling forces would allow therapeutic agents to counteract the TIFP prior to exerting onboard, oxygen sensor-based directional control towards the hypoxic areas. To be successful, such a robotic approach must be developed with a thorough knowledge of the main technological constraints, including those imposed by the microenvironments encountered during transit from the point of injection to the targeted treatment site. To better appreciate the challenges imposed by these constraints, various tumor microenvironments through which therapeutic nanorobotic agents would travel are briefly described.

## 2. Tumoral Microenvironments

**Figure 1:**
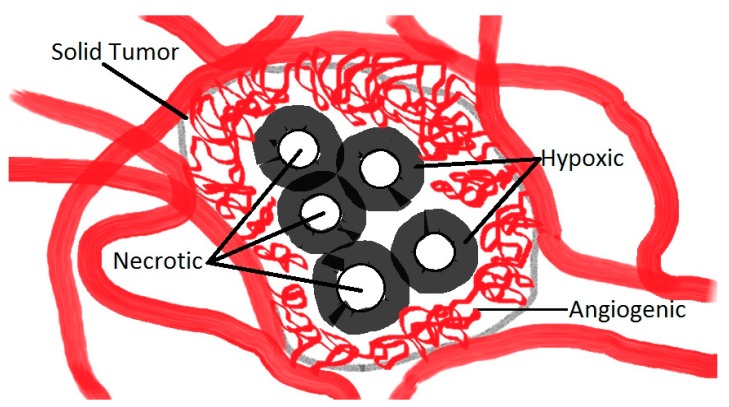
Simple schematic representing the main microenvironments of a solid tumor. Blood flow becomes negligible at the hypoxic-angiogenic transition due to the tumor interstitial fluid pressure (TIFP) which constitutes a barrier to the diffusion of conventional therapeutic agents towards the hypoxic regions. Oxygen concentration is maximal at the hypoxic-angiogenic transition and decreases gradually to zero (anoxic) past the hypoxic-necrotic boundaries.

Typically, the design of a robot should take into account the types of environments in which it will operate. This remains true for the microenvironments that must be transited in order to reach the tumoral regions to be treated. A simplistic description of these microenvironments is depicted in Figure 1. A solid tumor consists of tumor cells, interstitium (the space between cells in tumoral tissue), and the vasculature (the angiogenic capillary network). In the case of a peritumoral injection, the first tumor microenvironment that must be transited by therapeutic nanorobots is the angiogenic microenvironment (Figure 1). Several types of solid tumors are accessible to this type of therapy, e.g., colorectal and prostatic tumors. This first microenvironment can be described as a complex and chaotic capillary network with blood vessels of just a few micrometers (μm) in diameter. In this microenvironment, blood typically flows towards the tumor; however, in pre-existing microvascular vessels, blood flow will deviate from the tumor. In these regions, blood flows at various rates at approximately 0.3 mm/s in capillaries [8]. Therefore, the overall size of each agent cannot exceed a few micrometers in diameter to allow successful transit through the angiogenic network. Another critical aspect that may bring additional insights regarding the maximum permissible diameter of such a nanorobotic agent concerns the endothelial cells present on most if not all tumor vessels. In the tumoral environment, endothelial cells do not form the expected monolayer and thus do not have normal barrier function. Instead, these cells are disorganized, irregularly shaped and have loose interconnections and focal intercellular openings, which are responsible for much of the vessel leakiness observed in solid tumors. The diameter of these openings is typically less than 2 μm [9], emphasizing the need to maintain the maximum diameter of each navigable nanorobotic agent below 2 μm to enhance targeting in solid tumors. Navigating through these intercellular openings may prove to be critical for two main reasons. First, to increase the proportion of nanorobotic agents that enter the tumor’s interstitial space from the peritumoral injection site, the intercellular openings may provide an entryway to tumor vasculature, in contrast to healthy endothelium circumventing the tumor and being devoid of such openings. Second, once in the angiogenic network, such openings would provide entry points to the tumor interstitial space which must be traveled in order to reach hypoxic areas.

Hence, following transit through the angiogenic environment, such agents would be able to reach hypoxic and necrotic regions by entering the tumor interstitial fluid microenvironments. At the entrance of this microenvironment, therapeutic agents would experience an elevated TIFP which would increase the resistance to interstitial fluid flow and, in most cases, would lead to inadequate diffusion of non-propelled pharmaceutical agents. Therefore, propelled agents offer a real advantage compared to existing pharmaceutical agents, theoretically reaching deeper into the tumor microenvironments. Although the TIFP is known to be relatively constant inside interstitial tumor environments, the pressure gradient against human tumor walls can reach levels ranging typically from 10 to 40 mmHg (1 mmHg = 133.322 Pa = 1.33 × 10^−10^ N·μm^−2^) [10,11,12]. It is then not surprising that such relatively high pressure gradients impose serious constraints for the injection of micrometer-sized agents directly inside the tumoral volume. Indeed, a perforation of the tumor would not be clinically acceptable in most cases due to the increased risk of metastases. Alternatively, entering the tumor using the same physiological routes that permit passage of nutrients and oxygen through the angiogenic microenvironment would be a more suitable approach.

Once these agents have entered the tumor interstitial fluid microenvironment, the next challenge would be to move towards hypoxic regions. These regions are not necessarily located at the center of the tumoral volume but can be distributed at different locations inside the tumor. Since the exact locations of such hypoxic regions are unknown and not visible without sufficient spatial resolution using any existing medical imaging modalities alone, effective targeting in these critical regions is very challenging but necessary to achieve optimal therapeutic outcomes. Knowing that oxygen content in the interstitial fluid is decreasing gradually from the blood vessel capillaries that constitute the angiogenic network to ~0.5% in the hypoxic zones, to zero in the necrotic regions, suggests that an oxygen (O_2_) sensory system embedded in each navigable agent could become a critical addition. To be effective, such an O_2_ gradient detector should not only be embedded in each navigable agent, but the information being gathered should be used to influence the directional displacement of the agent. Once an oxygen threshold of ~0.5% is detected in hypoxic regions, the release of therapeutic payloads at optimal locations would assist in achieving the best therapeutic outcomes.

## 3. MC-1 Therapeutic Bacterial Nanorobots

To bypass current technological limitations, these therapeutic nanorobots were implemented by harnessing microorganisms possessing the required functionalities and specifications. For example, bacteria have been identified as potential alternatives to artificial constructs for targeting tumoral regions [13]. Particular species of bacteria can be highly suitable for tumor targeting due to their adequate dimensions, allowing them to transit the angiogenic network and the intercellular openings of the angiogenic vessels. Their self-powered capability avoids the requirement for inductive power in artificial structures, known to be a major constraint for miniaturization. Their embedded flagellated propulsion systems have also proven to be very effective in low-Reynolds-number hydrodynamic conditions as encountered in the tumor microenvironments. Furthermore, for some species, the ability to seek low oxygen concentrations with a threshold similar to the oxygen concentration encountered in the hypoxic regions of tumors is a critical feature, and not presently achievable for artificial nanorobots.

**Figure 2:**
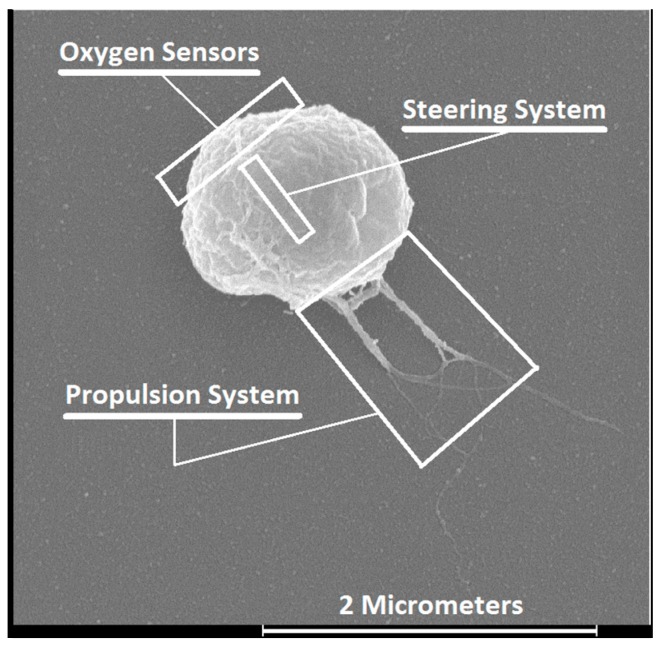
Main nanorobotic components or subsystems identified on a field-emission photograph of the MC-1 cell. The propulsion system consists of two bundles of flagella activated by rotary molecular motors with a design similar to the ones found in modern engineered electrical motors. Flagellated propulsion is known to be very effective in low-Reynolds-number hydrodynamic conditions. Computer-controlled direction is provided by an embedded steering system consisting of a chain of membrane-based magnetic nanoparticles acting as a miniature compass needle. Oxygen sensory capability can influence the motion of the cell towards the hypoxic regions when the torque induced on the steering system from a directional magnetic field becomes negligible (Adapted from [16].

In particular, the magnetotactic bacterium (MTB) strain MC-1 has been a serious candidate for implementing such a sophisticated cancer-fighting nanorobot [14] (Figure 2). Scanning Electron Microscopy (SEM) of the MC-1 bacterium shows the three main components or subsystems needed for an efficient nanorobot. By attaching various therapeutic payloads to the MC-1 cell surface, such as covalently bound nanoliposomes [15], such an agent could be considered as a general nanorobotic carrier in cancer therapy.

Magnetism is not influenced by variations in tissue density and can be generated at any depth in the human body by an external platform. Based on the location of the tumor typically determined using a medical imaging modality, MTBs [17] could be excellent agents to fulfill the role of therapeutic nanorobots guided by a relatively weak directional magnetic field to achieve effective targeting of solid tumors [14]. MTBs are flagellated cells that synthesize a chain of intracellular single magnetic domain nanoparticles within a lipid bilayer, known as magnetosomes [18,19]. The magnetosome chain acts as a miniature compass needle which, when a directional torque is induced from a relatively weak magnetic field, can serve as an embedded navigation or steering system to influence the directional swimming paths of the MTBs.

Non-pathogenic anaerobic bacteria that preferentially localize and proliferate in hypoxic regions of tumors and consume oxygen essential for tumor cell growth have been previously investigated as a cancer treatment [20] with mixed success. To increase therapeutic outcomes, these bacteria were subsequently investigated as delivery vehicles of conventional drugs, a strategy that has been referred to as combination bacteriolytic therapy (COBALT). However, a major drawback was the high dose required to induce a significant therapeutic effect resulting in high toxicity [21] as the bacteria circulated in the systemic blood network.

Since combining bacterial therapy with other conventional methods such as chemotherapy [21] or radiotherapy [22] by carrying the required therapeutic cargo will most likely be mandatory to achieve complete tumor consumption or at least to enhance the therapeutic outcomes, non-systemic magnetotaxis-based directional control to guide drug-loaded MTBs and especially the MC-1 cells close enough to the hypoxic regions, where oxygen gradients are present and can be detected, represents a key factor to maximize the therapeutic index. This can be done following a peritumoral injection. Once in the tumor interstitial fluid environment, the magnitude of the magnetic field could be set sufficiently low to switch from magnetotaxis- to aerotaxis-based displacement of MC-1 bacteria. Such a scenario could potentially enhance the therapeutic effects with minimal systemic toxicity.

The swimming velocity of MTB is also a critical factor. Based on initial observations, the lifetime of MTB in the human body is expected to be 30–40 min, providing a brief window of time for the intervention. The thrust force provided by two bundles of flagella allows the MC-1 MTB to swim at average velocities of ~200 μm/s with peak velocities reaching ~300 μm/s (in water at room temperature). These values represent approximately 10 times the velocity of many other known species of flagellated bacteria under the same environmental conditions. The spherical MC-1 bacteria have a diameter ranging from 1 to 2 μm, which may prove ideal for reaching tumor hypoxic areas. In addition, MC-1 MTBs are polar. Unlike axial MTBs, the cell swims persistently in one direction. This polarity can be set at the region to be treated and generated for tumor targeting by a special platform developed by our group and known as the magnetotaxis system [23]. For polar MTBs, the direction of the cells (north- or south-seeking) can be preprogrammed or preselected prior to injection. This is not the case for axial MTBs which swim along the magnetic field lines in either direction. Furthermore, MC-1 cells do not carry toxic genes and preliminary tests performed in mice suggest that they are non-pathogenic. Also, this species of bacteria has been successfully cultivated in the laboratory with highly repetitive and reliable propelling and magnetotactic behaviors, making them serious candidates for the production of bacteria-based therapeutics with highly predictable behaviors for control and targeting purposes. Finally, the microaerophilic response of MC-1 bacteria forces them to migrate towards ~0.5% oxygen levels which generally corresponds to the oxygen level expected at tumor hypoxic regions. Altogether, the MC-1 nanorobotic agent depicted in Figure 2 fulfills all the fundamental specifications needed to effectively target hypoxic regions in solid tumors. Since accurate magnetotaxis directional control and agglomeration of the MC-1 cells in a three-dimensional (3D) volume has already been demonstrated [23] using a prototype platform of the magnetotaxis system, the next objective was to validate its use and to determine the thresholds of the magnetic fields required to switch between magnetotaxis-based and aerotaxis-based displacement of MC-1 cells. Such data could then be used to finalize the specifications of a future clinical magnetotaxis platform capable of delivering therapeutic payloads to the hypoxic zones of solid tumors using MC-1 cells as carriers.

## 4. Theory of the Switch from Magnetotaxis- to Aerotaxis-Based Displacements

The motion of MC-1 bacteria in a natural environment is influenced by magneto-aerotaxis [24]. Cells will swim initially deeper and then back-and-forth while maintaining their orientation along the lines of the geomagnetic field ranging from 25 to 65 μT (0.25 to 0.65 Gauss) in order to remain in a layer of oxygen where the concentration is ~0.5%. Hence, by creating an artificial environment based on various magnitudes of magnetic field, several taxis-based directional control methods can be implemented [13], namely deterministic, semi-autonomous, environmental, simultaneous multi-taxes, and switched (time-multiplexed) multi-taxes.

Such magnetotaxis-based navigation control is possible due to the alignment of MC-1 magnetosomes (intracellular compartments, each with a single magnetic domain Fe_3_O_4_ nanoparticle) that create a cellular magnetic dipole. When exposed to a magnetic field, this can be described by the Langevin function as

(1) $$\cos\theta =L\;\left(\frac{m\mu_{0}H}{k_{B}T}\right)$$

In Equation (1), *θ* is the angle between the direction of the cell magnetic moment *m* and the ambient directional magnetic field *H* = *μ_0_^−1^·B*, where *B* is the magnetic field density and *μ_0_* is the permeability of free space. The Boltzmann constant and the temperature are represented by *k_B_* and *T*, respectively. According to Equation (1), the level of magnetotactic directional control characterized by the alignment of the cell in the applied directional magnetic field will be determined by the ratio of the interactive magnetic energy with the applied field (*mB*) to the thermal energy (*k_B_T*), the latter being the thermal forces associated with Brownian motion that tend to randomize the cell orientation unless, as in the case of an MC-1 cell, there is an oxygen gradient.

For instance, if the ratio *mB*/*k_B_T* is sufficiently high (but not too high as other phenomena will transpire and prevent a proper directional guidance of the MC-1 cells), the cell will be fully aligned along the magnetic field direction, *i.e.*, sufficient directional torque will be induced to fully control the swimming direction of the bacteria. This has been referred to as the deterministic mode. Similarly, if the magnitude of the directional magnetic field decreases gradually from that used for the deterministic mode, the ratio *mB*/*k_B_T* decreases as well and the movement of the bacteria becomes gradually less deterministic by responding less to magnetotaxis-based directional control. This phase defines the assisted (semi-autonomous) mode, with various levels of assistance coming from the external magnetotaxis platform. In this mode, the bacteria are still directed effectively towards a targeted location but are given more freedom to avoid physiological obstacles. The latter would be ideally used to initially target the tumoral volume.

Reducing the magnitude of the magnetic field further leads to simultaneous multi-taxes control in the form of magneto-aerotaxis, as observed in natural settings. For an applied magnetic field with a magnitude below the level of the geomagnetic field, the microaerophilic response of MC-1 becomes the only (or most predominant) factor influencing its displacement towards lower oxygen concentrations in the tumor, unless no oxygen gradients are present. In that case, the cell would move in a more randomized pattern. The strategy of exploiting magnetotaxis followed by aerotaxis directional controls for targeting hypoxic regions can be categorized as switched (time-multiplexed) multi-taxes directional control [13]. In this particular case, the use of an assisted or semi-autonomous magnetotaxis-based control allows the cells to deviate autonomously from physiological obstacles not visible by any external means, including by any medical imaging modalities. This is followed by aerotaxis, to allow the nanorobotic agents to navigate towards hypoxic areas. Here we refer to this as Magneto-Aerotaxis Navigation (MAN) if assisted by a modulated magnetic field to enhance the targeting ratio; otherwise, it is referred to as Magneto-Aerotaxis Targeting (MAT), where finding the appropriate path towards the tumor is done entirely by the bacteria.

The next critical phase in this research is to determine the ranges of magnetic field strengths that would enable different displacement control modes (magnetically-assisted followed by aerotaxis, targeting a 0.5% oxygen threshold) of MC-1 therapeutic nanorobots in order to describe the specifications for implementing MAN in a future magnetotaxis system.

## 5. MAN within Technological and Clinical Constraints

**Figure 3:**
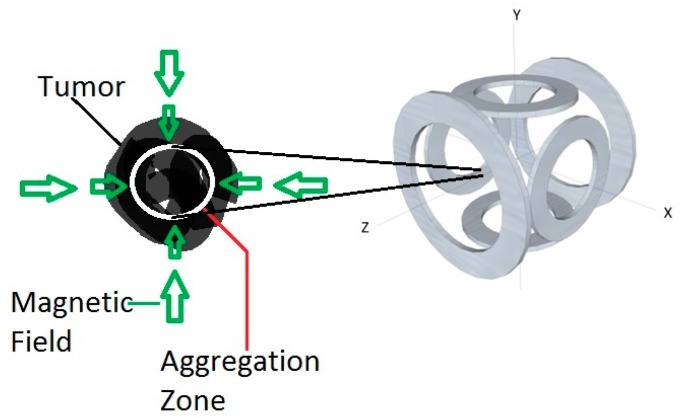
Basic operational principle of the magnetotaxis system aimed at exploiting the capability of MC-1 bacteria at delivering therapeutics to the hypoxic regions of solid tumors. The magnetic field strength is adjusted so that the outer ring of the aggregation zone will correspond to the switching threshold of MC-1 cells. Taking such a threshold into account, current circulating in the coils is adjusted so that the volume of the aggregation zone corresponds to the targeted volume inside the solid tumor.

The MAN principle, schematically represented in Figure 3, must take into account specific technological and clinical constraints, particularly the ones related to power, heat dissipation, overall dimensions of the tumors and related risks of metastasis.

The core of the magnetotaxis platform consists of a set of electromagnetic coils, represented in Figure 3 as three orthogonal pairs of electric coils positioned in a Maxwell configuration. Although other configurations could be envisioned, the depicted configuration has the advantage of being relatively simple and flexible. However, to scale it for providing an inner diameter sufficiently large for placing a human adult in it, another configuration is presently considered by our group. Independently of the configuration, using special time-varying magnetic field sequences [23], the first volumetric targeting zone called the aggregation zone (Figure 3) can be created at a specific 3D location. Typically this would correspond to the tumoral region in the patient. The directional magnetic field strength decreases as the distance from the coils increases. The magnitude of electrical currents circulating in the coils is therefore adjusted so that the outer boundary of the aggregation zone (Figure 3) would correspond to the field intensity where non-sufficient directional torques would be induced on the chain of magnetosomes in MC-1 cells. Aggregation would occur since any MC-1 MTBs exiting this 3D volume would be forced to re-enter such a volumetric aggregation zone due to a sufficiently high directional torque on the chain of magnetosomes. Inside the aggregation zone, magnetotaxis-based directional control is no longer possible due to a weak directional torque being induced on the chain of magnetosomes. Aerotaxis would take effect with the displacement of MC-1 cells guided by their microaerophilic behaviors.

Figure 3 represents a simplistic description of the method since the outer boundary of the aggregation zone cannot be described as a sharp line separating magnetotaxis from aerotaxis displacement behaviors. Instead, a larger layer of magnetotaxis-based displacement gradually fades to allow aerotaxis to take a greater role in the displacement behavior of MTBs. As such, the primary objective of this paper is to better understand the displacement behaviors of MC-1 cells within this outer boundary of the 3D aggregation zone.

One of the major technological considerations for implementing a clinical magnetotaxis platform is related to the overall size of the aggregation zone. New vessels (angiogenesis) are formed when the tumor grows beyond 1–2 mm in diameter due to the fact that the point diffusion of nutrients becomes rate-limiting for continued growth of the tumor [25]. Since MC-1 cells would travel along the same physiological routes used by nutrients entering the tumoral regions, a larger tumoral volume would most probably be preferable before injecting MC-1 cells in order to provide enough entry points [26] to yield a sufficiently high ratio of MC-1 cells in the tumor interstitial spaces. However, highly vascularized tumors may also have the potential to produce metastases by providing an efficient route of exit for tumor cells to leave the primary site and enter the bloodstream [27]. Therefore, interventions using MC-1 cells must be done before the tumor grows beyond a certain size where the risk of metastasis becomes significant. In renal carcinoma, for example, where MTB-based agents could be injected peritumorally, results suggested that the risk of metastatic disease is negligible in patients with tumors less than 3 cm [27]. Although more studies on various types of tumors may be required, such results suggest that the aggregation zone should not have a diameter larger than 3 cm and ideally be lower. Determining the best lower diameter for the aggregation zone to cover the whole tumoral region at the earliest time is difficult at the present time due to a lack of experimental data. Determination of the optimal overall size of the aggregation zones that will cover the whole tumor should also consider the fact that mutation begins when the tumor mass reaches ~1 mg or ~10^6^ cancer cells, while early clinical detection typically occurs when the tumor mass reaches ~1 gram or ~10^9^ cancer cells [27].

## 6. Experimental Results

The magneto-aerotactic behaviors of several species of MTBs, including MC-1 cells, have been recently reported [28]. The results provide detailed experimental data that are highly valuable to better understand and to predict the behavior of the MC-1 bacteria in the tumoral environments. One example is the oxygen concentrations being targeted by the MC-1 cells which are equivalent to those in hypoxic regions of solid tumors [29]. Since previous studies keep the magnitude of the magnetic field relatively constant, *i.e.*, without studying the effect of the magnitude of the magnetic field on the motion behavior of the MC-1 cells [28], the aim of the following complementary experiments was restricted to optical microscopy observations of MC-1 cells submitted to different magnitudes of directional magnetic fields to assess the ability of MC-1 cells to target hypoxic areas from an injection in the peritumoral region.

**Figure 4:**
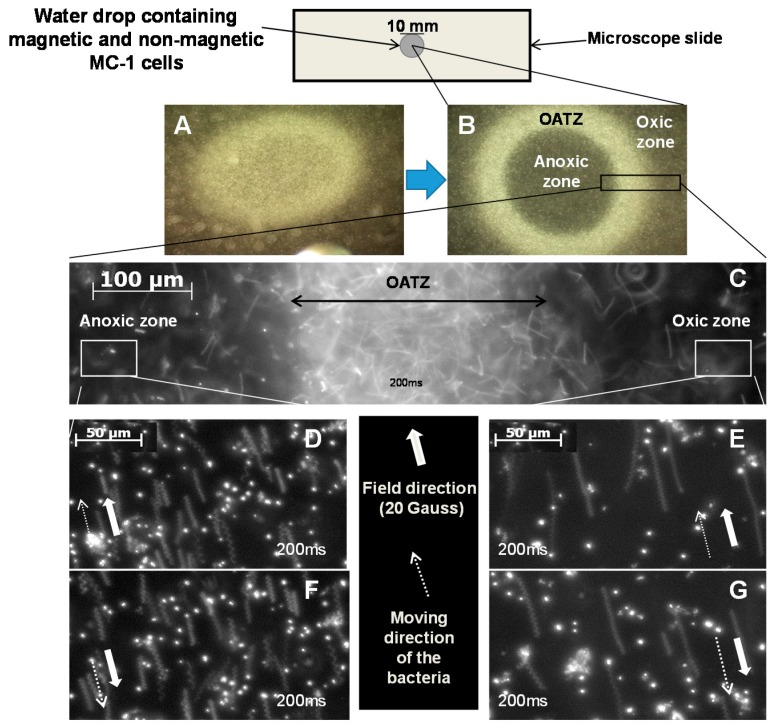
Motion behaviors of MC-1 bacteria. (**A**): Optical microscopy image of a mixture of magnetic MC-1 cells and non-magnetic mutants of the MC-1 cells immediately after mixing in a drop of water; (**B**): A ring of densely populated bacteria at the OATZ at ~0.5% O_2_ was initially formed in the presence of the geomagnetic field. The image shows MC-1 bacteria leaving the OATZ after a magnetic field of 20 Gauss is directed towards the anoxic and the oxic zones while the non-magnetic mutants remain in the OATZ; (**C**) Close-up of the section of (B) depicting a dense population of MC-1 cells in the OATZ with some magnetic bacteria already in the anoxic and the oxic zones immediately following the application of the directional magnetic field, successively directed towards the anoxic and the oxic zones; (**D**,**F**): Close-ups of a section of the anoxic zone depicting the magnetic MC-1 bacteria swimming in a new direction after the application of a 20 Gauss magnetic field; 200 ms exposure; (**E**,**G**): Close-ups of a section of the oxic zone depicting the magnetic MC-1 bacteria swimming in a new direction after application of a 20 Gauss magnetic field at lower velocities than the ones in the oxic zone, suggesting that MC-1 velocity decreases as the oxygen level decreases for the same directional magnetic field strength.

The total magnetic energy of the magnetosome chain takes into account the contribution of 10 to 15 closely spaced individual magnetite (Fe_3_O_4_) nanoparticles with a magnetic moment per MC-1 cell of *m* = 10^−16^ (A·m^2^). We show that a minimum magnetic field strength on the order of a few Gauss (e.g., ~5 Gauss (0.5 mT)) was sufficient to achieve a proper magnetotactic directional response of MC-1 bacteria along a predefined path. The results depicted in Figure 4 were obtained in an aqueous environment saturated with oxygen while 15 to 20 Gauss was ideal for oxic regions and to bypass the oxic-anoxic transition zone (OATZ) at ~0.5% O_2_.

For the experience depicted in Figure 4, the oxic-anoxic zone has been produced by a non-magnetic mutant of the MC-1 strain. This non-magnetic strain consumes oxygen in the central area of the microscope sample (Figure 4A,B) which results in the production of a circular band at the extremity of the oxic zone (Figure 4B). Observations of the motility of the MC-1 cells were done with a Zeiss AxioImager Z1 microscope with dark field. An AxioCamMR CCD camera (Zeiss, Göttingen, Germany) was used to acquire the images. The camera was directly attached to the microscope and had a resolution of 1388 pixels × 1040 pixels with a dynamic range of 12 bits. To get stable results, the exposure time of the camera was fixed to 200 ms (time frame of 200 ms) and the gamma camera was set to linear. The velocity of the bacteria cells was measured in culture media at room temperature. Since bacterial velocity was measured under oxic and anoxic conditions at the same time for any given sample, viability was not considered a key factor.

Experimental results are summarized in Figure 4. These results suggest that the magnetic MC-1 cells will most likely bypass hypoxic zones of tumors (<0.7% O_2_) when submitted to a directional magnetic field of 15 to 20 Gauss, with decreasing swimming velocity once past hypoxic areas and moving towards the necrotic zones (~0% O_2_). This is attributed to a lack of oxygen (below the threshold of ~0.5% O_2_). With a lower directional magnetic field between approximately 5 and 10 Gauss, micrographs (Image B) show that MC-1 cells will initially swim in the oxic zones at the same velocities as with a directional magnetic field of ~15 to 20 Gauss but momentarily remain stationary in the OATZ. These observations suggest that the movement of the MC-1 cells is reversed back-and-forth, similar to the polar magneto-aerotaxis behaviors observed in their natural environments. This swimming sequence in the OATZ may last up to a few minutes before the bacteria continue swimming past the OATZ towards anoxic areas if a magnetic field of 5 to 10 Gauss is maintained.

## 7. Discussion

The resulting magnetic field thresholds required to switch from magnetotactic-based to aerotactic-based displacement behaviors suggest that a magnetic field strength above ~5 Gauss should be directed towards the targeted region to be treated when the MC-1 bacteria carrying therapeutic payloads are located in the peritumoral regions. Indeed, below approximately 5 Gauss, it appears that magnetic MC-1 cells will remain in hypoxic regions of the tumor. This conclusion is based on observations showing that the bacteria remain in the OATZ for more than the expected survival period of 30–40 min of the MC-1 cells in the human body.

Due to the effect of a lower oxygen concentration on the velocity of the MC-1 MTBs, such directional magnetic fields should be increased to ~15 to 20 Gauss in order to eliminate (or at least negate) the effect of a lower oxygen concentration on the swimming velocity of the MC-1 cells. One should also keep in mind that the magnetic field threshold influencing each MC-1 cell within a large population will vary within a relatively narrow range of magnetic field strengths, and this has to be considered when interpreting the experimental results.

Based on these results, one of the main issues for clinical applications will be related to the optimal targeting approach to achieve maximum therapeutic efficacy considering the heterogeneous distribution of hypoxic areas within solid tumors. Indeed, for a given tumor volume, setting the outer boundary of the aggregation zone below ~5 Gauss around the tumor periphery may result in a large population of MC-1 bacterial carriers in the hypoxic areas, closer to the periphery of the tumor at the possible cost of a much lower population in the hypoxic areas located deeper in the tumoral volume. To target hypoxic areas located deeper in the tumor and bypass hypoxic areas near the periphery, the magnitude of the directional magnetic field may be gradually increased up to 15–20 Gauss near the tumor periphery. This targeting procedure could be repeated to bypass hypoxic areas near the periphery at the highest possible velocity. By doing so, the magnetic gradient generated by the magnetotaxis platform would have to be taken into consideration to ensure that the magnitude of the magnetic field is well below ~5 Gauss in the regions to be targeted. Diminution of the magnetic field to bypass pre-targeted hypoxic areas may also be a concern, especially for magnetic fields between ~5 to 10 Gauss, as MC-1 cells may temporarily remain in intermediate hypoxic areas, extending the time required to reach deeper hypoxic regions. On the other hand, this could also help to achieve a better distribution of the MC-1 cells throughout hypoxic regions inside the tumoral volume. Further experiments are needed to confirm this. Ideally, one should be able to decrease the diameter of the aggregation zone well below the total diameter of the tumoral volume, but this will likely impose serious constraints on the specifications of a potential human-scaled magnetotaxis system in terms of power and heat dissipation. In the case where all the tumoral volume must be targeted by MC-1 cells, it is anticipated that the aggregation zone should be enlarged to allow sufficient entry points through the angiogenic networks while allowing the targeting of sufficiently small tumors before the occurrence of metastases. However, a smaller aggregation zone, targeting smaller tumors in order to prevent metastases, would require more power to generate the appropriate magnetic fields. This additional power requirement places constraints not only on power delivery but also on power dissipation.

A spatial-based targeting approach pointing to the same 3D location of the aggregation zone (Figure 3) could be implemented in a more affordable clinical magnetotaxis platform using MC-1 bacteria for tumor size of 3 cm. This would reduce the risk of metastasis according to the study [24] on the risk of metastasis *versus* tumor sizes. For smaller tumors, more sophisticated spatial-based targeting sequences pointing to several predetermined targeted sites surrounding the tumor mass could be applied. A time-based targeting instead of a spatial-based targeting approach can also be considered where a directional magnetic field pointing towards the tumoral volume could be applied for a predetermined amount of time prior to removing (switching off) the magnetic field. This approach could yield good targeting efficacy in hypoxic regions of solid tumors while enhancing the distribution of the therapeutics in the hypoxic areas due to variations of the swimming velocities among MC-1 cells. However, if too much time would be allowed for the MC-1 cells to swim in a given direction, a proportion of the population of bacteria could bypass the tumoral mass. This may increase the systemic toxicity of MC-1 nanorobotic therapy and raise clinical concerns. However, despite the technological limitations, the distribution and connectivity of hypoxic regions within tumors could potentially help achieve a suitable distribution of MC-1 cells throughout hypoxic regions of the tumors. Further studies will help determine the best interventional strategy and take into account the displacement behaviors of MC-1 cells described here.

## 8. Conclusions

The magneto-aerotactic displacement behavior of the self-propelled microaerophilic MC-1 bacterium is potentially ideal for transporting therapeutic payloads to the hypoxic zones of solid tumors following peritumoral injections. As such, the swimming response of these agents to various magnitudes of directional magnetic fields becomes a key aspect for the implementation of a successful targeting strategy. Here, we identify the appropriate ranges of the directional magnetic field to guide MC-1 cells towards the tumoral volume by exploiting their magnetotaxis displacement behavior. This is followed by an autonomous aerotactic-based displacement towards the lower oxygen concentration of hypoxic zones. Determining the ranges of the magnetic field will help establish the specifications of future clinical platforms capable of exploiting the displacement behaviors of MC-1 cells, maximizing the therapeutic effects while minimizing systemic toxicity and more efficiently targeting the hypoxic areas of solid tumors.
